# Neonatal Whisker Trimming Impairs Fear/Anxiety-Related Emotional Systems of the Amygdala and Social Behaviors in Adult Mice

**DOI:** 10.1371/journal.pone.0158583

**Published:** 2016-06-30

**Authors:** Hitomi Soumiya, Ayumi Godai, Hiromi Araiso, Shingo Mori, Shoei Furukawa, Hidefumi Fukumitsu

**Affiliations:** Laboratory of Molecular Biology, Department of Biofunctional Analysis, Gifu Pharmaceutical University, Daigakunishi, Gifu, Japan; Radboud University Medical Centre, NETHERLANDS

## Abstract

Abnormalities in tactile perception, such as sensory defensiveness, are common features in autism spectrum disorder (ASD). While not a diagnostic criterion for ASD, deficits in tactile perception contribute to the observed lack of social communication skills. However, the influence of tactile perception deficits on the development of social behaviors remains uncertain, as do the effects on neuronal circuits related to the emotional regulation of social interactions. In neonatal rodents, whiskers are the most important tactile apparatus, so bilateral whisker trimming is used as a model of early tactile deprivation. To address the influence of tactile deprivation on adult behavior, we performed bilateral whisker trimming in mice for 10 days after birth (BWT10 mice) and examined social behaviors, tactile discrimination, and c-Fos expression, a marker of neural activation, in adults after full whisker regrowth. Adult BWT10 mice exhibited significantly shorter crossable distances in the gap-crossing test than age-matched controls, indicating persistent deficits in whisker-dependent tactile perception. In contrast to controls, BWT10 mice exhibited no preference for the social compartment containing a conspecific in the three-chamber test. Furthermore, the development of amygdala circuitry was severely affected in BWT10 mice. Based on the c-Fos expression pattern, hyperactivity was found in BWT10 amygdala circuits for processing fear/anxiety-related responses to height stress but not in circuits for processing reward stimuli during whisker-dependent cued learning. These results demonstrate that neonatal whisker trimming and concomitant whisker-dependent tactile discrimination impairment severely disturbs the development of amygdala-dependent emotional regulation.

## Introduction

Sensory defensiveness is a negative reaction to one or more types of sensation and is often associated with neurodevelopmental disorders such as autism spectrum disorder (ASD) and fragile X syndrome [1]. Tactile hyporesponsiveness is strongly associated with social and communication impairments in ASD patients [2]. Emotional memories associated with tactile perception are also important for attachment in infancy, defined in rodent studies as seeking proximity to and maintaining contact with the dam when pups are upset or threatened [3, 4]. This attachment is an early primitive social behavior; therefore, early tactile sensory defensiveness is likely to influence the development of neural circuits related to emotional and social behaviors, but this remains to be determined.

Whiskers are one of the most highly developed tactile organs in mice and serve as an important communication tool during neonatal development [5, 6]. Whisker trimming is frequently observed in laboratory mice associated with social hierarchy as socially dominant mice trim the whiskers of inferiors [5, 7]. In neonatal rats, the tactile information from whiskers (together with olfactory cues) is necessary to receive milk from the dam [8] and communicate with their siblings [9]. Tactile perception from whiskers, particularly during the neonatal period, might thus be critical for the development of social behaviors in rodents.

Whisker-specific tactile perception modules in the somatosensory cortex of rodents are organized as “barrel cortex” [10]. The barrel cortices receive input from thalamic afferents that specify the typical barrel pattern in cortical cyto-architecture and function within the first few postnatal days [11–14]. The critical period is postnatal days 10–14 (P10-14) in rodents for the functional maturation of neurons in the somatosensory area related to whiskers (the barrel field, S1BF) [15, 16]. Long-lasting changes in whisker receptive fields are produced by tactile deprivation during the early postnatal period [17–20]. Specifically, whisker trimming in neonates leads to hyper-responsiveness of cortical barrel neurons due to wide-ranging and permanent abnormalities in the local inhibitory circuitry, even after whiskers fully regrow [17]. Whisker trimming during the neonatal period also impairs whisker-dependent discrimination and associated behaviors in adulthood [21–23]. Rats subjected to whisker trimming during P0–P3 showed shorter crossable distances in the gap-crossing test, higher exploratory activity, and increased social interaction times, even after full whisker regrowth by the time of testing [22]. In addition, whisker trimming of rats during P9–P20 decreased emotional reactivity, such as freezing duration and flight reaction, at P25 [24]. These findings suggest that early postnatal tactile experience is critical for the anatomical and functional maturation of the related somatosensory system [25, 26] and might also affect the formation of emotional systems related to social behavior. However, the variations in whisker trimming duration, the developmental stage at which whisker trimming was performed, and the time point of behavioral assessment across these studies [22, 24] make it difficult to establish a precise association between early tactile deficits and sustained changes in social behavior. In the present study, mouse whiskers were bilaterally trimmed for 10 days after birth (BWT10 mice) to disturb the critical period of S1BF maturation (including thalamocortical afferents, barrel formation, and cortico-cortical projections [14, 16]). Adult BWT10 mice were tested for learning of whisker-dependent discrimination tasks and social behaviors. Following stress and behavior testing, mice were also examined for c-Fos expression in the amygdala as a marker of neural activity because amygdala circuits are critical for processing emotional information.

## Materials and Methods

### Animals

All experiments were approved by the Animal Research Committee of Gifu Pharmaceutical University, Gifu, Japan, and conducted in accordance with the National Institutes of Health guidelines on animal care. All efforts were made to minimize the number of animals used and their suffering.

Pregnant ddY mice (embryonic day 17–18; E17-E18) were purchased from Japan SLC (Shizuoka, Japan) and monitored at 24-h intervals to establish time of birth. The day of birth is defined as postnatal day (P) 0.

### Sensory deprivation

In the trimming group (BWT3 and BWT10), pups were restrained by hand and all whiskers were trimmed daily to within 1 mm of the skin for 3 and 10 days, respectively, beginning at P1. In the control group, pups were restrained by hand but whiskers were left untrimmed. The pups were allowed to mature without further intervention except for weekly cage cleaning. The female pups were sacrificed by pentobarbital overdose (100 mg/kg) at 3 weeks (P3W), and only age-matched P8W–P9W males were used in experiments. All whiskers of both BWT3 and BWT10 mice regrew and were of the equivalent length to controls by P8W–P9W.

### Behavioral tests

#### Gap-crossing test

The apparatus consist of two custom-built black Plexiglas platforms (6 cm wide × 15 cm long × 20 cm high) connected by two identical 2-cm diameter pipes that together form a runway with a manually adjustable gap distance. Two 5 × 5 cm walls were attached to both sides of the platform upper surfaces at the facing ends for the mice to easily recognize the gap distance. Gap-crossing procedures were conducted as follows. The mice were food deprived for 24 h before testing. To prevent the use of visual information, the tests were conducted in a darkened room. The experimenter kept a red-filtered flashlight on hand, but it was not shined on the mice, so the ambient light exposure was less than 1 Lux. The mice were initially trained to find a reward (a small food pellet) at the opposite end of the connected runway. After this training phase, a gap was inserted in the runway and was widened in 0.5-cm intervals. If the mice crossed the gap to obtain the food reward within 2 min, it was considered a successful trial. The gap was widened until the mice would no longer step across it within the 2-min period. This protocol was repeated two times in succession to obtain an average maximum gap width that the individual mouse would cross.

#### Three-chamber social interaction test

The testing apparatus consisted of a 30 cm wide × 60 cm long × 20 cm high Plexiglas box divided into three chambers as described previously [27, 28]. The mice could move between the chambers through a small opening (6 × 6 cm) in each chamber divider. Plexiglas restraining cylinders were placed in each of the two side chambers, one of which contained a probe mouse. Numerous holes in the cylinders enabled contact between the test and probe mice. Mice to be tested were placed in the center chamber and allowed 5 min to explore the entire box, after which an unfamiliar, same-sex probe was placed in one of the two restraining cylinders. Test mouse movements were recorded using a video camera positioned above the Plexiglas box. The time spent in the social and opposite (empty) chamber was measured.

#### Social dominance tube test

Social dominance between control and BWT10 mice was measured by the tube test as previously described [29]. Briefly, the apparatus was a transparent Plexiglas tube 30 cm in length with a 3-cm inner diameter. The tube could be separated into sections by two removal gates 13 cm from each end. The diameter was sufficient to permit only one mouse to walk through without reversing direction. Prior to the test trial, each mouse was released at either end of the tube without the gates down for 2 min. After this 2-min habituation period, two unfamiliar mice of approximately the same age (P8W–P9W) and matched as closely as possible for body size and weight (37–40 g), one control and one BWT10, were simultaneously released at the opposite ends of the tube. The dominant mouse was considered the one that advanced across the midline or the one that pushed the other mouse out of the other end within 2 min. Each mouse was matched with two different opponents with an at least 5-min interval between trials.

#### Eight-arm radial maze

The custom-built apparatus consisted of a center platform with eight radiating arms, each 5 cm wide × 25 cm long × 15 cm high, numbered 1 to 8. The arm floors were made of black Plexiglas and surrounded by clear 6-mm thick Plexiglas walls. First, a food deprivation schedule was administered to reduce body weight to 85% of baseline. For this purpose, feeding was restricted to 2 h per day for 2 consecutive days. One day before the actual training began, groups of four mice were habituated to the apparatus by placing them at the center and allowing free exploration for 5 min both with and without a bait placed at the arm ends. In the baited condition, the mice were allowed to retrieve the bait, a single 3-g food pellet placed in a food cup. Following habituation and shaping, each animal was individually placed in the center of the maze and trained once a day for 12 consecutive days. The inner wall surfaces of four arms (numbers 1, 3, 5, and 7) were covered with a wire net (1.4 cm mesh; 21 cm long × 7 cm high), and the food cups in these four arms were baited with a single 10-mg food pellet per cup for each daily training trial, while an empty food cup was placed at the ends of the other four arms without wire nets (numbers 2, 4, 6 and 8). Each mouse was allowed to freely explore until it had taken all the pellets or 5 min had elapsed. Measures were made of the ratio of entries into the net-covered/baited arms to total arm entries (ratio of net arm choice) and number of arm revisits. At 2 h after the task, the brains of the mice were processed for immunocytochemistry.

### Stress load procedure

The behavioral stress protocol was based on a previous report [30]. Briefly, the mice were individually placed on an elevated circular platform (6 cm diameter × 25 cm high) for 30 min; the mice showed freezing, defecation, and urination under this stress condition. At 2 h after stress, the mice were killed and tissues were prepared for histochemistry.

### Tissue preparation

Brains were processed as described previously [28]. Briefly, brains were removed after intracardial perfusion with 4% paraformaldehyde in phosphate-buffered saline (PBS) and post-fixed in the same fixative overnight. The brain tissues were immersed in PBS containing 20% (w/v) sucrose for cryoprotection and then frozen in an embedding compound (Sakura Finetechnical, Tokyo, Japan). Coronal serial sections of 30-μm thickness were prepared on a cryostat (Leica, Germany, model CM 1800), stained as described below, and mounted on gelatin-coated slides (Matsunami, Osaka, Japan).

### Immunostaining and Nissl staining

Immunohistochemical analyses were performed using previously described procedures with a slight modification [31]. In brief, free-floating serial coronal sections (30 μm) were collected in PBS. For c-Fos immunohistochemistry, every third section was processed as follows. Sections were incubated overnight in 0.1 M Tris-HCl (pH 7.4) containing 0.3% H_2_O_2_ and 0.3% Triton X-100, washed three times with Tris-buffered saline (TBS), and blocked for 30 min with 2% (w/v) Block Ace (Dainippon Sumitomo Pharma Co. Ltd, Osaka, Japan) dissolved in TBS. Blocked sections were incubated overnight at 4°C with anti-c-Fos (1:3000; Santa Cruz Biotechnology, Inc., Santa Cruz, CA). After three washes in TBS, the sections were incubated for 3 h with biotinylated anti-rabbit goat IgG (1:1000; Vector Laboratories, Burlingame, CA), washed again in TBS, and reacted with ABC solution (Elite ABC, Vector Laboratories) at 4°C overnight. After three washes in TBS, the sections were incubated in 0.1 M acetate buffer (pH 6.0) containing 0.05% 3,3′-diaminobenzidine tetrahydrochloride solution (Dojindo Laboratories, Kumamoto, Japan), 2.5% ammonium nickel sulfate (Nacalai Tesque, Kyoto, Japan), 0.2% β-D-glucose (MP Biomedicals, Santa Ana, CA), 0.04% ammonium chloride (Wako Pure Chemical Industries, Ltd., Osaka, Japan), and 0.0005% glucose oxidase (Toyobo, Osaka, Japan). After washing, the sections were mounted on gelatin-coated slides, cleared with xylene, and coverslipped using Eukit (O. Kindler, Freiburg, Germany). The adjacent series of sections was used for Nissl staining with 0.1% thionin, dehydrated in an ascending ethanol series, cleared in xylene, and coverslipped with Eukit. For estimating the number of c-Fos positive cells, stained cells were counted in each brain region and cortical layer specified using the criteria described below.

### Quantitative studies on c-Fos-positive cells

Three to five sections from each stained series were chosen for the quantification of c-Fos-positive neuron number in the prefrontal areas and amygdala, and five sections were chosen for the quantification of stained c-Fos-positive neurons in S1BF. The sections of choice were those closest to dorsal–ventral level interaural 6.02 mm (for prefrontal area), 2.22 mm (for amygdala), and 2.86 mm (for S1BF) according to the atlas of Paxinos and Franklin [32]. Only the left hemisphere from these sections was quantified. For each brain section, the number of c-Fos-immunopositive cells in a given brain structure was counted, divided by the area occupied by that structure (in mm^2^), and expressed as positive-cell density. For cortical areas, the entire depth of the cortical field was included in a particular section. The borders of the cortical areas and subcortical nuclei were determined using adjacent Nissl-stained sections. These borders were drawn by an investigator blind to the experimental group assignment of the animals and reviewed by a second investigator. The area was measured by ImageJ (National Institutes of Health; NIH).

### Statistical analyses

**Fig 1:**
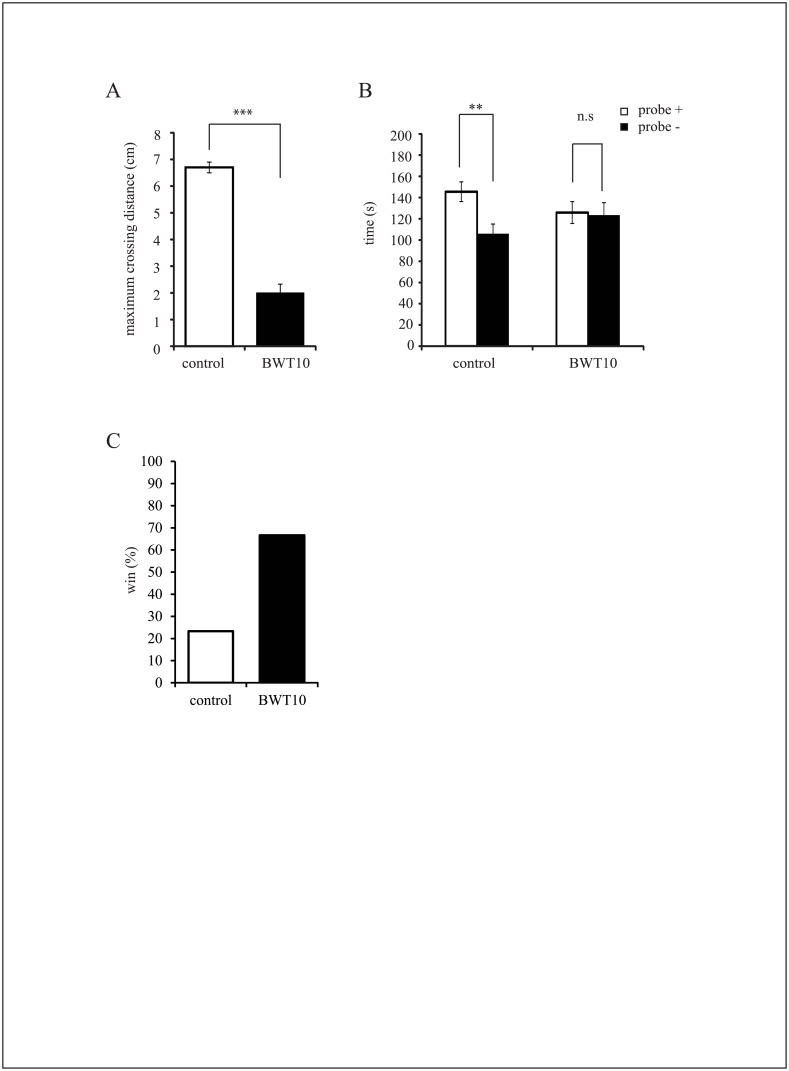
Neonatal whisker trimming disrupted tactile sensory performance and social interactions of mice in adulthood. **A**, Adult BWT10 mice showed a significant deficit in gap-crossing performance compared to controls as evidenced by a shorter mean maximum crossable distance. Values are expressed as the mean ± SE. ***p < 0.001 versus control, Student’s *t*-test; n = 5 for control mice, and n = 6 for BWT10 mice. **B**, In the social interaction test, control mice showed a strong preference for the social side chamber containing a probe mouse, whereas adult BWT10 mice showed no such preference between the social and empty chambers. All values are expressed as the mean ± SE. **p < 0.01, n.s., no significance versus social chamber, Wilcoxon’s test; n = 23 for control mice, and n = 22 for BWT10 mice. **C**, In most trials, control mice retreated (“lost”) when BWT10 and control mice faced each other in the tube test apparatus. Values are expressed as the percentage of wins. *p < 0.05, significantly different from chance 50:50 outcome, χ test; n = 15 for control mice, and n = 15 for BWT10 mice.

Data are presented as the mean ± standard error of the mean (S.E). Group means from behavioral data were compared by Student’s *t*-test. Paired histological data were also compared by Student’s *t*-test. Multiple group means were compared by one-way analysis of variance (ANOVA) with Tukey’s post hoc analysis or two-way ANOVA with Bonferroni’s post hoc analysis. The social interaction test (Figs 1B, S1 and S2A) was analyzed by Wilcoxon’s test. The social dominancy in the tube test (Figs 1C and S2B) were analyzed using the χ^2^ test.

## Results

### Neonatal whisker trimming resulted in adult social behavior deficits

To examine the effect of neonatal whisker trimming (P1–P10) on adult whisker function, we compared the control mice to the BWT10 mice in the gap-crossing test, which requires the mice to evaluate a gap and make a decision (to cross or not) based on tactile information from the whiskers. The gap-crossing test was performed in the dark so that the mouse relied only on whisker-dependent tactile information to locate a target platform across a gap. The mean maximum distance of the gap that the control mice successful crossed was 7.0 cm (Fig 1A) compared to less than 2.0 cm for the adult BWT10 mice (Fig 1A). This result indicates that neonatal whisker trimming impaired tactile perceptivity of the adult BWT10 mice to detect the target platform over a longer gap distance even though all whiskers had regrown to same length as that of the controls by the time of testing.

Maternal–infant separation during early postnatal development is a highly stressful situation for mice that has long-term influences on adult social behaviors [33–37]. Because tactile contact plays important roles in maternal–infant interactions, we examined the effects of whisker trimming at birth on several social behaviors in the adult mice. In the three-chamber social interaction test, the control mice spent significantly more time in the “social” side chamber containing a conspecific than in the “nonsocial” empty side chamber, whereas the adult BWT10 mice showed no preference for the social chamber (Fig 1B). Next, we investigated social dominance between the adult control and BWT10 mice. In the tube test, the more dominant mouse in a social hierarchy shows greater aggression and forces its opponent out of a tube when both are placed at opposite ends and must then use the other end for escape [29]. The adult BWT10 mice won significantly more head-to-head confrontations (20/30, 66.7%) against the control mice than expected by chance (χ2 = 5.933 *p* = 0.015) (Fig 1C). Therefore, whisker trimming at birth altered social behavior as well as tactile perception in adulthood, even though whiskers were fully regrown by the time of the tests.

### Neonatal whisker trimming altered stress-induced c-Fos expression pattern in the amygdala and frontal cortex

**Fig 2:**
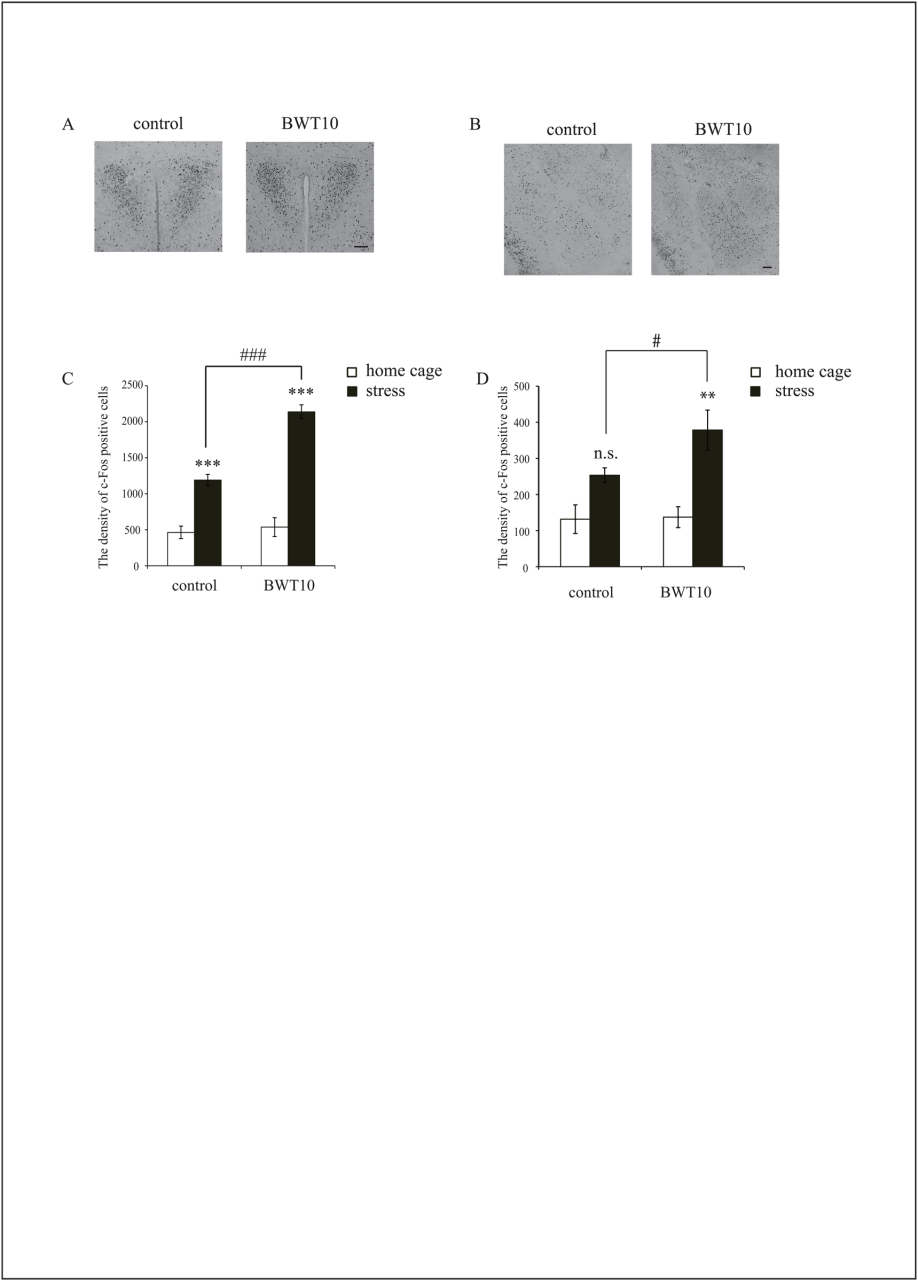
Neonatal whisker trimming altered stress-induced c-Fos expression pattern in the emotional system of adult mice. Distribution of c-Fos-expressing neurons in PVN (**A**) and the amygdala (**B**) of mice 2 h after being placed on a small elevated platform. The graphs show the density of c-Fos-positive neurons in PVN (**C**) and the amygdala (**D**). Scale bar, 100 μm. Values are expressed as the mean ± SE. ***p < 0.001, n.s., no significance versus home cage, #*p* < 0.05, ###*p* < 0.001 versus control mice, One-way ANOVA with Tukey’s post hoc test; n = 4 for control and BWT10 mice in their home cages, and n = 5 per group under height stress.

**Fig 3:**
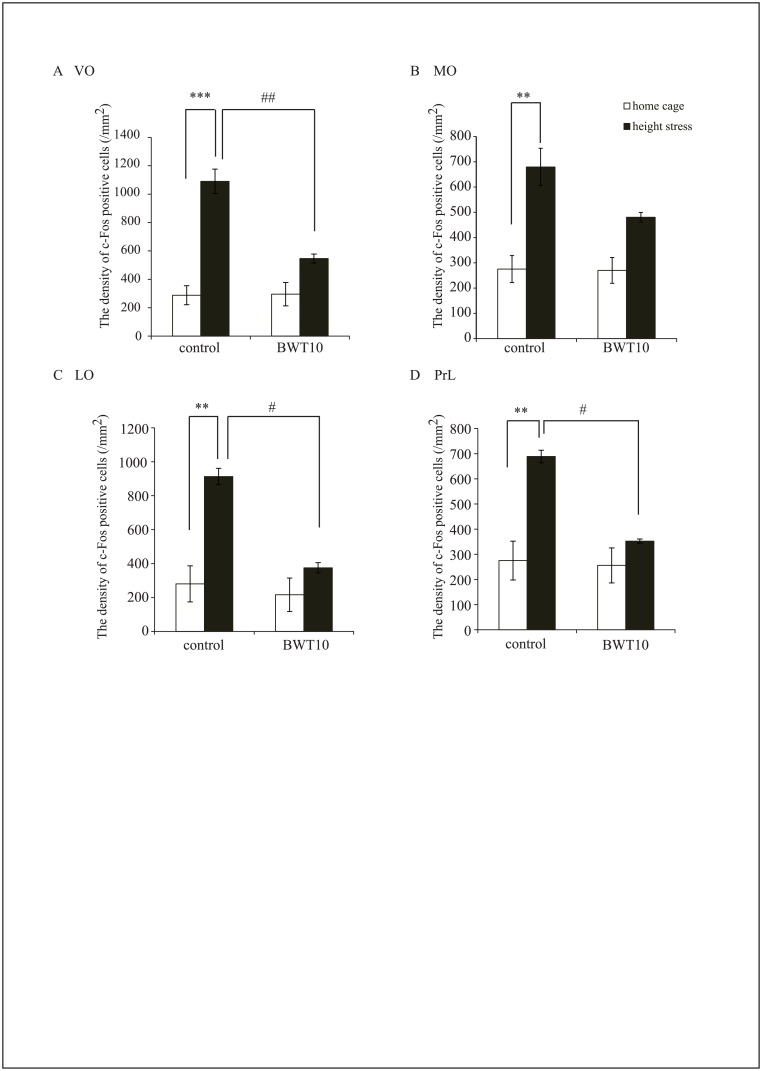
Neonatal whisker trimming altered stress-induced c-Fos expression in the frontal cortex of adult mice. Quantitative analysis revealed significantly higher c-Fos-positive cell density following elevated platform stress in the frontal cortex of control mice, while c-Fos-positive density in the frontal cortex of BWT10 mice was not significantly altered by this stress (**A–D**). Values are expressed as the mean ± SE. **p < 0.01, ***p < 0.001 versus home cage, #*p* < 0.05, ##*p* < 0.01 versus control mice, One-way ANOVA and Tukey’s post hoc test; n = 4 for control and BWT10 mice in their home cages, and n = 5 per group under height stress.

Emotional regulation has important implications for social behavior as well as social contacts dependent on multimodal perception, including whisker tactile perception [5, 38]. To evaluate if neonatal whisker trimming affects the development of the emotional system, we examined stress-induced neural activation in several brain regions associated with emotional processing, the basolateral amygdala, paraventricular nucleus (PVN), and prefrontal cortex [medial orbital (MO), ventral orbital (VO), and prelimbic cortex (PrL)] following exposure to elevated platform stress. In the control mice, c-Fos-positive cells were significantly increased 2 h after stress in PVN and prefrontal cortex and there was a trend for increased expression in the amygdala (Figs 2 and 3). However, stress-induced c-Fos expression in the amygdala and PVN of the adult BWT10 mice was significantly higher than of the control mice, whereas expression in the prefrontal cortex of the BWT10 mice was not altered by the stress (Figs 2 and 3). These data suggest that the adult BWT10 mice show aberrant stress-induced neuronal hyperactivity within the emotional system, including amygdala and PVN.

### Neonatal whisker trimming did not affect whisker-cued memory or related reward processing

**Fig 4:**
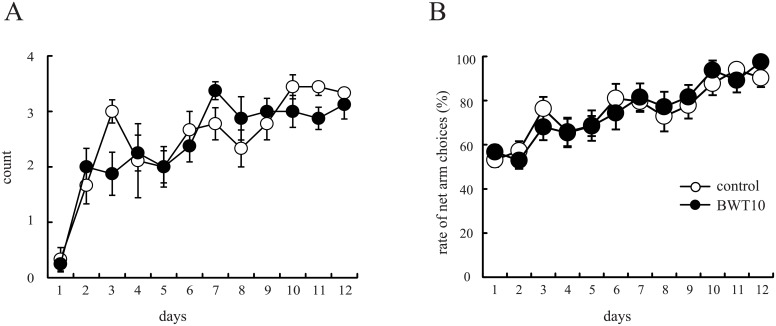
Neonatal whisker trimming did not affect learning in a whisker-cued memory task. The net arm choice among the first four entries (**A**) and the ratio of net arm choice (**B**) increased with daily trials in both control and BWT10 mice. No significant differences were observed in these parameters between control and BWT10 mice (two-way ANOVA with Bonferroni’s test). n = 9 for control mice, and n = 8 for BWT10 mice.

The amygdala plays important roles not only in emotional regulation but also in reward-contingent behavior. Indeed, reward-driven neuronal activity in the amygdala is known to modulate memory formation during radial maze appetitive training in mice [39–41]. Thus, to clarify whether neonatal whisker trimming affects reward-motivated whisker tactile perception and memory, we analyzed daily performances of the adult control and BWT10 mice during the learning of an eight-arm radial maze task under conditions requiring the detection of whisker cues for reward. Four arms of the maze were cued with wire nets and baited at the ends, while the other four arms contain no tactile cues and were never baited. In this apparatus, the mice had to learn and memorize the relationship between tactile cue and reward and the spatial relationship among cued/baited and uncued/unbaited arms. Both groups showed selective entering into the net-covered arms across trials (one trial per day for 12 consecutive days). The ratio of net arm choice also increased over training days and plateaued at the same level by day 10 in both groups (Fig 4), indicating that the BWT10 mice could learn and memorize the association between the tactile cue and reward (baited arms) as efficiently as the control mice. Thus, neonatal whisker trimming did not impair tactile perception based reward-driven memory formation.

**Fig 5:**
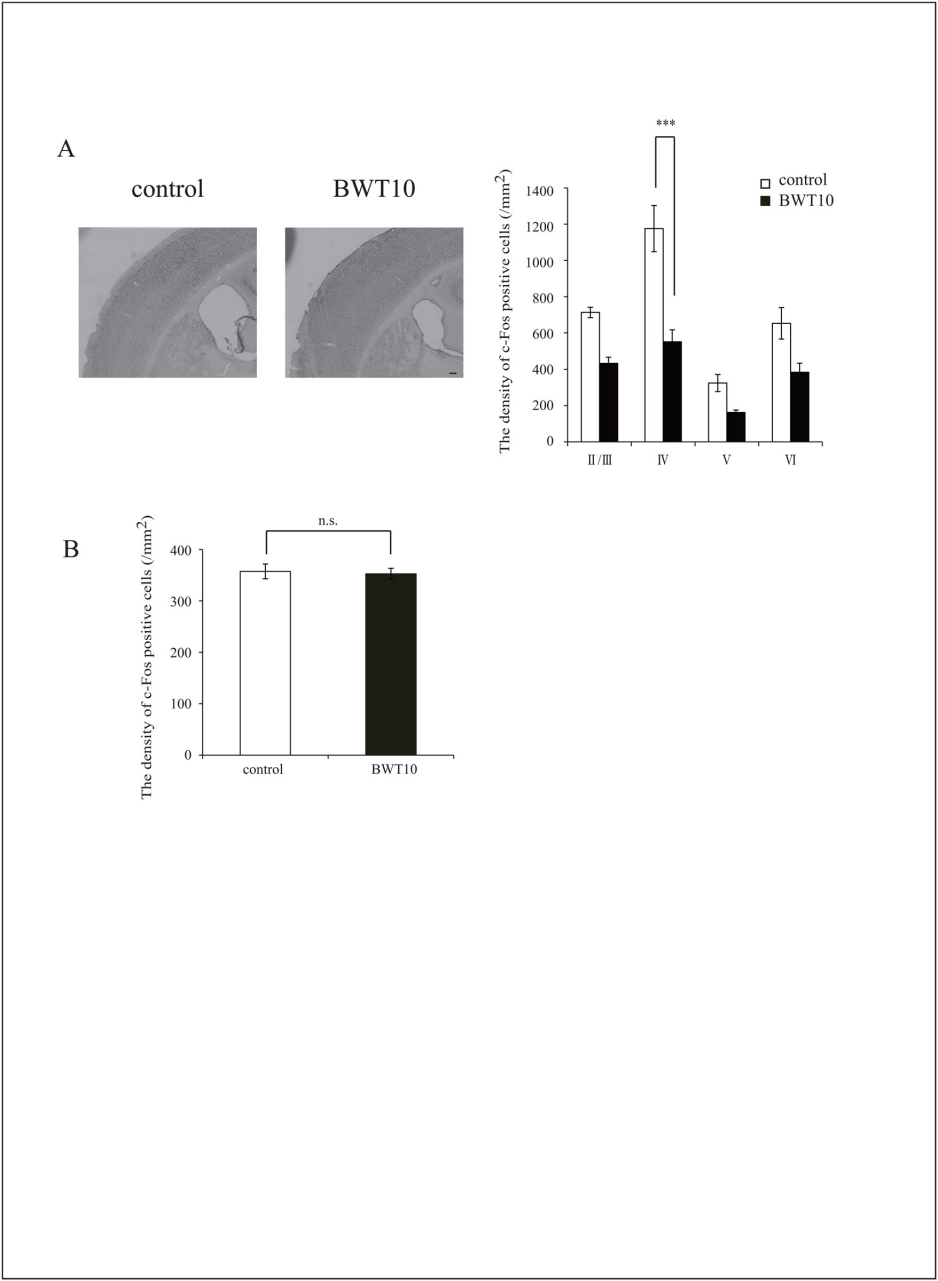
Neonatal whisker trimming altered c-Fos expression in S1BF but not in the amygdala of adult mice following learning of whisker-cued memory task. **A**, Distribution of c-Fos-expressing cells in the somatosensory cortex 2 h after the net-guided radial maze task. The graphs show the density of c-Fos-positive cells in S1BF. The density of c-Fos-positive cells in S1BF layer IV of BWT10 mice was significantly lower than in control mice. Scale bar, 100 μm. **B**, There was no difference in c-Fos-positive cell density in the amygdala between control and BWT10 mice. Values are expressed as the mean ± SE. ***p < 0.001, One-way ANOVA and Tukey’s post hoc test; n = 5 for control mice, and BWT10 mice.

Next, to address whether neonatal whisker trimming affects neuronal activity in structures engaged by this task, we examined c-Fos expression in S1BF, the prefrontal cortex, and the amygdala 2 h after the 12^th^ training trial. The density of c-Fos-positive cells significantly increased in S1BF layer IV, amygdala, and prefrontal cortex of both groups after training. However, the increase and final density in S1BF layer IV were significantly smaller in the BWT10 mice than in the control mice (551.3 ± 66.4 vs. 1175.8 ± 127.2, respectively; Fig 5A), while increases were comparable between the groups in the amygdala (Fig 5B) and prefrontal cortex (S1 Table). Nissl staining revealed that the total number of neurons in the regions examined was comparable in both the groups (S2 Table). Taken together with the results from the gap-crossing test, these results indicate that neonatal whisker trimming impairs the development of circuits for processing whisker tactile discrimination in the somatosensory system but not neuronal circuits for reward processing in the amygdala. Both systems were activated during learning of the whisker-cued maze task, but as discussed below, neuronal function in the S1BF would likely be less important for performance than in the gap-crossing task.

## Discussion

In this study, we found that whisker trimming for 10 days after birth caused long-lasting dysfunction of whisker-dependent tactile perception as revealed by the gap-crossing test (Fig 1A), as well as abnormalities in social-related behaviors such as social interaction and social dominance (Fig 1B). Furthermore, neonatal whisker trimming severely affected the development of amygdala circuitry related to fear/anxiety processing as shown by altered c-Fos expression patterns following the height stress compared with that in controls (Fig 2). In contrast, whisker trimming did not alter amygdala circuits related to reward processing as revealed by unchanged c-Fos expression patterns compared with that in controls following whisker-dependent cued training in the radial maze task (Fig 3). These results indicated that the neonatal suppression of tactile perception and experience due to whisker trimming impair the development of emotional systems, leading to long-lasting changes in social behavior.

To what extent did neonatal whisker trimming affect the development of whisker-dependent tactile perception and cognitive systems? In the gap-crossing task, the mean maximum gap distance was only 2.0 cm for the BWT10 mice. For successful gap crossing, mice must decide whether they are able to cross the gap based of whisker information; however, at such short distances, the mice can find the target platform by touching it with their nose as well as with their whiskers [42]. Thus, BWT10 mice may have severe difficulties perceiving the gap distance and/or the shape of the target platform using their whiskers. On the other hand, BWT10 mice could learn the radial maze tactile-cued task with their whiskers, whereas normal adult mice failed to learn this maze task when all whiskers were trimmed prior to the first daily trial and once every three trials thereafter (Soumiya et al., unpublished data). Furthermore, S1BF neuronal activity as estimated by the number of c-Fos-positive cells was significantly upregulated after the 12 daily trials in BWT10 mice but to a significantly lesser degree than that in the control mice. These results suggest that limited sensory processing is sufficient for BWT10 mice to learn the net-guided radial maze task but not the gap-crossing test. Thus, the most plausible explanation is that BWT10 mice lose higher-order sensory and/or sensory-motor integration for whisker-dependent tactile perception. Indeed, it has been suggested that the integration of sensory and motor information is required for learning the gap-crossing test because the information for performance is derived from individual whiskers moving and touching objects synchronically or independently [43–45].

Whisker-dependent tactile perception is also important for the social behavior of mice. Adult mice that had their whiskers trimmed immediately prior to testing exhibited reduced aggressive social behaviors against strangers or intruders [6, 46]. Similarly, adult mice with whiskers plucked prior to the test showed no preference for the social chamber in the three-chamber social interaction test (S1 Fig). In the case of mice subjected to whisker trimming as neonates, preference for the social chamber was maintained only in those subjected to bilateral whisker trimming for just 3 days after birth (BWT3) (S2A Fig), whereas the BWT10 mice showed no preference for the social chamber, although their whiskers had fully regrown by the time of testing. Moreover, the BWT10 mice showed social dominancy against controls, while the BWT3 mice did not (Figs 1 and S2B). Similar observations were previously reported in laboratory rats, subjected to whisker trimming during P0–P3 [22]. The difference between the BWT10 and BWT3 mice could result from the duration of sensory deprivation and concomitant effects on neural circuit development. Although there may be multiple causes underlying these abnormalities in BWT10 mouse social behavior, we suggest that impaired neonatal tactile experience and social interaction induces stress that may disrupt the development of emotional systems. This hypothesis is strongly supported by the greater neuronal activation in the amygdala/PVN induced by height stress in the BWT10 mice than in the control mice (Fig 2). Furthermore, social isolation from the dam in the early postnatal period causes emotional dysfunction and abnormalities in social behaviors later in life as well as impaired whisker perception [33–38].

Amygdala circuits play key roles in processing different information related to fear/anxiety and rewarding/aversive outcomes, which in turn modulate sensory perception, memory formation, and social behavior [47]. Each neuronal system within the amygdala comprises distinct neuronal subtypes within amygdala and could activate individually. Indeed, some amygdala neurons excited by aversive cue never respond to a reward cue during associative learning in the rat amygdala [48]. Comparing to the relative control, neurons in the amygdala of the BWT10 mice were found to be hyper-reactive against the height-stress but respond normally for the reward processing in the radial maze task (Figs 2B and 5B). These data indicated that the fear/anxiety-related circuit would be more vulnerability against neonatal whisker trimming than the reward relation in the amygdala.

Tactile defensiveness, defined as extreme sensitivity or aversive responsiveness to touch that would be benign to most people (e.g., light touch or clothing texture) is a common feature of neurodevelopmental disorders such as ASD and fragile X syndrome [49–51]. The tactile perception system is the earliest to develop among sensory systems. During infancy and early childhood, tactile perception provides important information from the outside world and an opportunity for environmental interactions, particularly with the mother [1]. Therefore, genetic and environmental causes of tactile defensiveness and not only neonatal whisker trimming are likely to impair attachment formation, an early primitive social behavior. Further studies are necessary to elucidate the molecular mechanisms underlying the abnormal development of sensory and emotional circuits caused by neonatal whisker trimming; however, we believe that such studies will have important implications for understanding the pathogenesis of neurodevelopmental disorders.

## Supporting Information

S1 Fig Acute whisker removal altered the social behavior of adult mice. All whiskers of P8W ddY male mice were plucked using a pair of tweezers under pentobarbital anesthesia (50 mg/kg) one day before the three-chamber social interaction test. The control mice received only pentobarbital anesthesia. Whisker-plucked mice did not show a preference for the social side chamber containing a probe mouse. All values are expressed as the mean ± SE. ***p < 0.001, Wilcoxon’s test; n = 6, control mice; n = 6, test mice.
(EPS)

S2 Fig Neonatal whisker trimming for 3 days after birth did not alter social behavior in adulthood. A, Similar to age-matched control mice, adult BWT3 mice showed a strong preference for the social side chamber containing a probe mouse in the three-chamber social interaction test (Fig 1B). All values are expressed as the mean ± SE. ***p < 0.001 versus social chamber, Wilcoxon’s test; n = 27, for control mice; n = 27, for BWT10 mice.. B, In the tube test, BWT3 mice did not show social dominance against control mice. Values are expressed as the percentage of wins. *χ*^2^ = 1.93, *p* = 0.164, *χ*^2^ test; n = 15 for control mice, and n = 15 for BWT3 mice.
(EPS)

S1 Table The number of c-Fos-positive cells in the frontal cortex of mice after the net-guided radial maze task. (DOCX)

S2 Table The number of Nissl-positive cells. (DOCX)
