# Supplementary material for: Neonatal Whisker Trimming Impairs Fear/Anxiety-Related Emotional Systems of the Amygdala and Social Behaviors in Adult Mice
Source: PLoS One. 2016 Jun 30;11(6):e0158583. doi: 10.1371/journal.pone.0158583 (PMC4928826; doi:10.1371/journal.pone.0158583)
Supplement: S1 Table — (DOCX) [file pone.0158583.s003.docx]

S1 Table. The number of c-Fos positive cells in the frontal cortex of mice after net-guided radial maze task.

|  | Control | BWT10 | *p* value |
| --- | --- | --- | --- |
| VO | 943.0 ± 60.4 | 1008.4 ± 107.5 | 1.00 |
| MO | 859.8 ± 38.5 | 839.0 ± 49.7 | 0.97 |
| LO | 567.2 ± 46.2 | 578.1 ± 102.4 | 0.99 |
| PrL | 780.9 ± 75.3 | 752.5 ± 68.6 | 1.00 |

Values expressed as the mean ± SE. The data was analyzed by One-way ANOVA and Tukey’s post hoc test; n = 4 for control mice, and n = 6 for BWT10 mice.
