# Supplementary material for: Neonatal Whisker Trimming Impairs Fear/Anxiety-Related Emotional Systems of the Amygdala and Social Behaviors in Adult Mice
Source: PLoS One. 2016 Jun 30;11(6):e0158583. doi: 10.1371/journal.pone.0158583 (PMC4928826; doi:10.1371/journal.pone.0158583)
Supplement: S2 Table — (DOCX) [file pone.0158583.s004.docx]

S2 Table. The number of Nissl positive cells.

|  |  | Control | BWT10 | *p* value |
| --- | --- | --- | --- | --- |
| S1BF | layer II /III | 2439.6 ± 126.2 | 2541.5 ± 123.7 | 1.00 |
|  | layer IV | 3552.4 ± 175.7 | 3692.6 ± 84.9 | 0.99 |
|  | layer V | 2177.2 ± 118.3 | 2044.5 ± 69.8 | 0.99 |
|  | layer VI | 2794.5 ± 74.0 | 2656.6 ± 87.1 | 0.99 |
| prefrontal cortex | VO | 2929.8 ± 181.0 | 2816.0 ±129.0 | 1.00 |
|  | MO | 2243.9 ± 73.0 | 2284.7 ± 84.3 | 1.00 |
|  | LO | 2167.4 ± 185.8 | 2302.9 ± 50.5 | 0.99 |
|  | PrL | 1840.3 ± 83.6 | 2009.8 ± 75.9 | 0.96 |
| amygdala |  | 1324.0 ± 21.1 | 1331.1 ± 27.2 | 0.85 |

Values expressed as the mean ± SE. The data was analyzed by One-way ANOVA Tukey’s post hoc test for somatosensory cortex, n=5 for control mice, and n=6; for BWT10 mice; for prefrontal cortex, n=4 for control mice, and n=6 for BWT10 mice.. The number of Nissl positive cells in amygdala was analyzed by Student’s t-test
